# Two Cases of Catheter-Related Bloodstream Infection: A Previously Unreported Complication of Long-Term Ritodrine Tocolysis

**DOI:** 10.7759/cureus.101468

**Published:** 2026-01-13

**Authors:** Hiroshi Mori

**Affiliations:** 1 Department of Obstetrics and Gynecology, Kenwakai Otemachi Hospital, Kitakyushu, JPN

**Keywords:** adverse drug events, gram-negative bacteremia, hospital-acquired infection, multidrug-resistant pathogen, pregnant female

## Abstract

Prolonged use of ritodrine hydrochloride (RT) for threatened preterm labor is discouraged in Western countries due to a lack of proven efficacy and severe maternal risks; however, it remains common in Japan. We report two rare cases of catheter-related bloodstream infections (CRBSI) in pregnant women undergoing long-term peripheral-line RT infusion. The first case involved *Stenotrophomonas maltophilia *bacteremia, a multidrug-resistant pathogen rarely reported in pregnancy. Despite limited antibiotic options, the patient recovered and delivered a healthy infant. The second case involved *Achromobacter xylosoxidans *bacteremia, treated successfully with ceftazidime, resulting in favorable maternal and neonatal outcomes. Both pathogens are opportunistic, typically affecting immunocompromised hosts or patients with indwelling devices, and their occurrence in otherwise healthy pregnant women underscores the risks of long tocolysis. These cases highlight CRBSI as a potentially life-threatening complication of long-term intravenous RT and emphasize the need to carefully balance its use against adverse outcomes.

## Introduction

Long-term tocolysis with ritodrine hydrochloride (RT) has not been proven effective in preventing preterm birth [1,2]. The United States Food and Drug Administration (FDA) advises against prolonged RT use in threatened preterm labor (TPL) because of maternal risks such as pulmonary edema, granulocytopenia, rhabdomyolysis, and venous thrombosis [3]. Nevertheless, long-term intravenous RT remains common in Japan, where the relatively low preterm birth rate compared with Western countries is often cited in support [4]. The largest concern regarding such a non-evidence-based treatment is the possible increase in adverse outcomes for women, including venous thromboembolism resulting from a prolonged duration of bed rest and intravenous infusion [3].

Catheter-related bloodstream infection (CRBSI) is also well known as a complication associated with long-term venous catheter placement. In recent years, CRBSI and central line-associated bloodstream infection caused by central venous catheters (CVCs) have been increasingly recognized worldwide as major clinical problems. Although the incidence of peripheral venous catheter (PVC)-related bloodstream infections, which are frequently used in the treatment of TPL, is relatively low, with a range of 0-2.2%, the absolute number of cases is not negligible due to the extremely high global use of PVCs [5]. Factors that increase the risk of infection include prolonged catheter dwell time, emergency insertion, and inadequate management of the insertion site. Coagulase-negative staphylococci and *Staphylococcus aureus* are the predominant pathogens, with *S. aureus* accounting for a higher proportion of PVC-related infections in particular. In addition, PVC-related infections are characterized by a shorter interval between catheter insertion and onset of infection compared with CVC-related infections [6].

We identified two pregnant women with CRBSI caused by a multidrug-resistant pathogen. Both women were undergoing "long-term colysis" treatment using intravenous RT for TPL at our hospital. To our knowledge, there are no reports of CRBSI occurring in pregnant women who received long-term tocolysis using a peripheral-line administered intravenous RT. We present reports on two patients with rare but life-threatening complications, detailing their clinical courses, test results, and pregnancy outcomes.

## Case presentation

Case 1

A 29-year-old Japanese woman (gravida 2, para 1) had been receiving maternal medical check-ups regularly at our hospital since her natural conception. She was a never-smoker and non-drinker, and had no significant past medical history. She visited our hospital with complaints of uterine contractions at 325/7 gestational weeks (GW). She was diagnosed with TPL due to frequent uterine contractions and cervical shortening (18 mm), so she was admitted to the hospital.

We initiated continuous peripheral-line intravenous treatment with RT at a dose of 50 µg/min. Uterine contractions resolved promptly after treatment initiation, and no additional cervical shortening occurred. Therefore, continuous infusion therapy at a flow rate of 50 µg/min was continued. Gram staining of vaginal secretions revealed an intermediate Nugent score. Treatment with metronidazole vaginal tablets for one week improved the condition. On day 11 of hospitalization, she developed a fever of 39.3°C accompanied by shaking chills. She was fully conscious, and her physical exam was remarkable for pulse 130 beats/minute, blood pressure 121/68 mmHg, respiratory rate 22 breaths/minute, and oxygen saturation 100% on room air.

The peripheral line insertion site on her left forearm was red and hardened, so we replaced the peripheral catheter on the right forearm. The patient reported that this location was painful. We ruled out lower respiratory tract infections and urinary tract infections (UTIs). Nonstress test (NST) showed a reassuring fetal heart rate pattern with no uterine contractions. Antigen testing for COVID-19, influenza A, influenza B, and group A *Streptococcus *(GAS) was negative. Although her quick sequential organ failure assessment (qSOFA) score was only one point, she presented with shaking chills, prompting a blood draw for cultures. With invasive GAS (iGAS) infection, a life-threatening infection during pregnancy, in mind, antibiotic therapy was initiated with sulbactam/ampicillin (12 g/day) and clindamycin (1200 mg/day). After treatment began, her body temperature decreased, and her chills improved.

On day 12 of hospitalization, gram-negative bacilli suspicious for *Pseudomonas aeruginosa *were detected in blood cultures within 24 hours, and the antibiotic therapy was changed to meropenem (3 g/day). On day 15, the causative bacteria were identified as* Stenotrophomonas maltophilia*. We show the susceptibility testing profile of the isolated bacteria from blood culture in Table 1. The isolated *S. maltophilia* strain was multidrug resistant, and all susceptible antibiotics (minomycin, levofloxacin, ciprofloxacin, sulfamethoxazole-trimethoprim) were antimicrobial agents whose use during pregnancy is generally discouraged. Although the antimicrobial susceptibility test showed that meropenem was not susceptible to the causative bacteria, it was judged to be effective clinically, and treatment was continued. On the same day, blood was drawn again for culture testing, but it turned positive the next day.

**Table 1:** Susceptibility testing profile of the bacterial isolate (Case 1) I, Intermediate; MIC, Minimum Inhibitory Concentration; NA, Not Applicable; N/R Not reported; R, Resistant; S, Susceptible

Antibiotics	MIC	Interpretation
Ampicillin (ABPC)	>16	NA
Piperacillin (PIPC)	N/R	-
Cefazolin (CEZ)	>16	NA
Cefotiam (CTM)	>16	NA
Cefotaxime (CTX)	>2	NA
Ceftriaxone (CTRX)	>2	NA
Ceftazidime (CAZ)	>8	NA
Cefepime (CFPM)	>16	NA
Cefaclor (CCL)	>16	NA
Cefcapene pivoxil (CFPN-PI)	>1	NA
Cefmetazole (CMZ)	>32	NA
Flomoxef (FMOX)	>32	NA
Imipenem/cilastatin (IPM/CS)	>2	NA
Meropenem (MEPM)	>2	NA
Aztreonam (AZT)	>8	NA
Sulbactam/ ampicillin (SBT/ABPC)	>16	NA
Sulbactam/ cefoperazone (SBT/CPZ)	N/R	-
Tazobactam/ piperacillin (TAZ/PIPC)	N/R	-
Gentamicin (GM)	>8	NA
Amikacin (AMK)	>32	NA
Minocycline (MINO)	≤2	S
Levofloxacin (LVFX)	≤0.5	S
Sulfamethoxazole-trimethoprim (ST)	≤40	S
Fosfomycin (FOM)	>16	NA

We stopped using RT on day 15 of hospitalization because we did not detect a shortening of the length of the uterine cervix. Although blood cultures remained positive, her general condition was good, and the pain at the intravenous insertion site had improved. The meropenem treatment was completed after one week. She was discharged on the 18th day of hospitalization (351/7 GW), and closed outpatient monitoring of the mother and fetus was continued. Delivery was scheduled for during the 36 GW. Postpartum treatment with a susceptible antimicrobial agent was planned.

**Figure 1:**
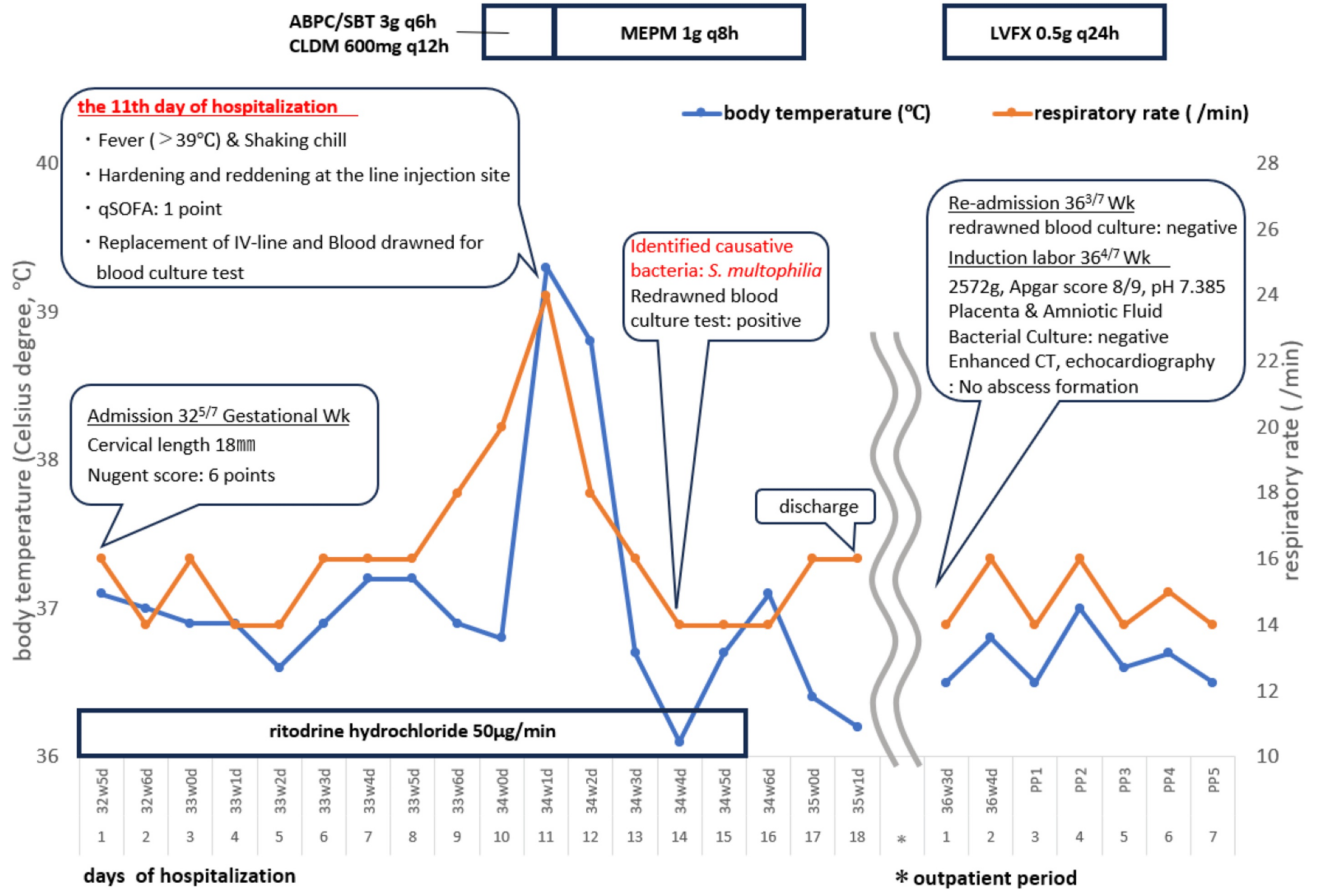
Clinical course in Case 1 ABPC/SBT, Ampicillin/sulbactam; CLDM, Clindamycin; GAS, Group A *Streptococcus*; IV, Intravenous; LVFX, Levofloxacin; MEPM, Meropenem; qSOFA, quick Sequential Organ Failure Assessment; Wk, Week

She was readmitted at 363/7 GW to induce labor during the preterm period. Blood cultures were drawn upon admission, but they turned negative for 48 hours. The day after readmission at 364/7 GW, she went into labor and delivered a healthy newborn male weighing 2,572g with an Apgar score of 8 at the first and 9 at the fifth minute. Bacterial culture tests of the placenta and amniotic fluid were negative, and no abscesses were detected in the postpartum maternal whole-body contrast-enhanced CT scan or echocardiography. She was treated with levofloxacin (0.5 g/day) intravenous infusion for seven days, starting immediately after delivery, and was discharged. Both the mother and the baby are progressing well and do not require additional treatment. A timeline of the patient's treatment course is summarized in Figure 1.

Case 2

A 26-year-old Vietnamese woman (gravida 1, para 0) had been receiving regular maternal checkups at our hospital since her natural conception. She was a never-smoker and a non-drinker, with no significant past medical history. She presented with uterine contractions at 27 3/7 GW. She was diagnosed with TPL due to frequent uterine contractions and cervical shortening (16 mm) and was admitted to the hospital.

Continuous intravenous tocolysis with RT at a dose of 50 µg/min via a peripheral line was initiated. Uterine contractions resolved promptly after treatment initiation, and no further cervical shortening occurred. Based on her clinical course, cervical incompetence was excluded. Continuous infusion therapy was continued at 50 µg/min. Gram staining of vaginal secretions revealed a normal Nugent score. On hospital day 10, she developed a fever of 39.3 °C with shaking chills. She was fully conscious, with physical findings of pulse 110 beats/minute, blood pressure 108/74 mmHg, respiratory rate 20 breaths/minute, and oxygen saturation 100% on room air.

The left forearm peripheral line insertion site was red and indurated, so the catheter was replaced on the right forearm, which the patient reported as painful. NST showed an elevated baseline fetal heart rate without uterine contractions. FilmArray® respiratory panel (bioMérieux, Inc., Lyon, France) multiplex polymerase chain reaction (PCR) testing of a nasopharyngeal swab was negative for all 20 pathogens (17 viruses and three bacteria) [7]. Lower respiratory infection and UTI were excluded. Although her qSOFA score was one, blood cultures were obtained due to the presence of shaking chills. Despite a negative GAS antigen test from a pharyngeal swab, empiric antibiotic therapy with sulbactam/ampicillin (12 g/day) and clindamycin (1200 mg/day) was initiated, assuming iGAS infection. After treatment, her fever resolved, chills improved, and baseline fetal heart rate normalized.

**Figure 2:**
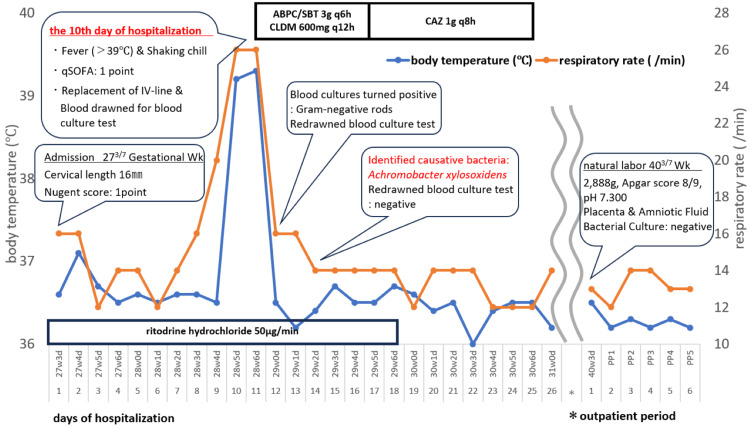
Clinical course in Case 2 ABPC/SBT, Ampicillin/sulbactam; CAZ, Ceftazidime; CLDM, Clindamycin; GAS, group A Streptococcus; IV, intravenous; qSOFA, quick Sequential Organ Failure Assessment; Wk, weeks.

On hospital day 13, blood cultures grew gram-negative bacilli. As both mother and fetus remained clinically stable, repeat blood cultures were drawn the same day and remained negative after 48 hours. RT was discontinued on day 14, as no further cervical shortening was detected. On day 15, the causative organism was identified as *Achromobacter xylosoxidans*. The antimicrobial susceptibility profile of the isolate is shown in Table 2. The strain was not multidrug resistant, and de-escalation therapy with ceftazidime was administered for 10 days. She was discharged on hospital day 26. Her pregnancy remained uncomplicated after discharge, and at 40 3/7 GW she went into labor and delivered a healthy male newborn weighing 2,888g, with Apgar scores of 8 at one minute and 9 at five minutes. A summary of her clinical course is shown in Figure 2.

**Table 2:** Susceptibility testing profile of the bacterial isolate (Case 2) I, Intermediate; MIC, Minimum Inhibitory Concentration; NA, Not Applicable; N/R Not reported; R, Resistant; S, Susceptible

Antibiotics	MIC	Interpretation
Piperacillin (PIPC)	≤8	S
Ceftazidime (CAZ)	≤4	S
Cefepime (CFPM)	16	I
Imipenem/cilastatin (IPM/CS)	≤1	S
Meropenem (MEPM)	≤1	S
Doripenem (DRPM)	N/R	-
Aztreonam (AZT)	>16	R
Sulbactam/ cefoperazone (SBT/CPZ)	≤16	NA
Tazobactam/ piperacillin (TAZ/PIPC)	≤8	S
Gentamicin (GM)	8	I
Tobramycin (TOB)	8	I
Amikacin (AMK)	32	I
Minocycline (MINO)	≤2	S
Levofloxacin (LVFX)	≤0.5	S
Ciprofloxacin (CPFX)	0.5	S
Sulfamethoxazole-trimethoprim (ST)	≤40	S
Fosfomycin (FOM)	>16	NA
Colistin (CL)	N/R	-

## Discussion

*S. maltophilia* is an opportunistic gram-negative bacillus increasingly recognized as a nosocomial pathogen [8,9]. Though usually avirulent in healthy adults, its capacity to colonize respiratory epithelia and medical devices facilitates infection in hospitalized patients [10,11]. Risk factors include exposure to broad-spectrum antibiotics [12,13]. Only two pregnancy-associated cases have been reported: a CRBSI during hyperemesis gravidarum [14] and a UTI in a woman with a nephrostomy tube [15]. Case 1 in our report represents the first well-documented *S. maltophilia* CRBSI in a pregnant woman treated with RT. 

*A. xylosoxidans *is another emerging opportunistic pathogen. It causes bacteremia mainly in patients with malignancies or chronic conditions requiring vascular access [16,17]. Only one pregnancy case has been described in Japan, though the source was unclear [18]. Case 2 in our report is the first reported instance, to the best of our knowledge, of *A. xylosoxidans *infection associated with RT in a pregnant woman.

It is noteworthy that all published cases of CRBSI from peripheral catheters in pregnant women, including the two reported by us, have come from Japan [14,18]. Prolonged tocolysis, combined with extended bed rest and catheter use, may predispose to such infections [3].

Our observations highlight that CRBSI caused by unusual pathogens can occur in otherwise healthy pregnant women undergoing long tocolysis. These infections are clinically significant because therapeutic options are limited during pregnancy, particularly for multidrug-resistant organisms. However, based on these cases, it is impossible to determine whether this life-threatening event is attributable to the drug itself or to peripheral catheter placement.

Therefore, the decision to continue prolonged intravenous RT must carefully balance potential benefits against maternal risks, including rare but life-threatening infections.

## Conclusions

These two cases illustrate that life-threatening CRBSI can arise in healthy pregnant women receiving long-term peripheral-line RT tocolysis. The involvement of uncommon and, in one case, multidrug-resistant gram-negative pathogens underscores the clinical challenges associated with treating severe infections during pregnancy, when antimicrobial options are restricted.

Although our observations do not clarify whether the infections were triggered by the drug itself or by the prolonged use of peripheral catheters, they highlight a critical safety concern associated with non-evidence-based prolonged tocolysis. Therefore, decisions regarding extended intravenous RT must be made with careful consideration of maternal risks, including rare but potentially fatal bloodstream infections.
